# Clinical characteristics and prognostic analysis of calcified glioblastoma

**DOI:** 10.1097/MD.0000000000047069

**Published:** 2026-01-16

**Authors:** Mengda Li, Juntao Li, Yongji Guo, Zhixiao Li, Guanzheng Liu, Xingyao Bu, Chunxiao Ma

**Affiliations:** a Department of Neurosurgery, People’s Hospital of Henan University, Zhengzhou, Henan Province, China; b Department of Neurosurgery, Henan Provincial People’s Hospital, Zhengzhou, Henan Province, China; c Department of Neurosurgery, People’s Hospital of Zhengzhou University, Henan Province, Zhengzhou, China.

**Keywords:** calcification, clinical features, glioblastoma, imaging, pathology, prognosis

## Abstract

**Rationale:**

Glioblastoma (GBM) is a common malignant intracranial tumor. There are a few clinical studies focusing on GBM with calcification. Based on diagnosed cases from our cohort and related literature reports, we investigate its clinical characteristics and diagnosis-treatment strategies.

**Patient concerns:**

A retrospective analysis was conducted on the clinical data of 4 patients with pathologically confirmed GBM with calcification admitted between 2017 and 2023. In addition, 5 cases with complete clinical data were identified through a literature review, yielding a total of 9 cases. Based on these cases, we analyzed and discussed the clinical features, treatment strategies, and prognostic outcomes of GBM with calcification in combination with the published literature.

**Diagnoses:**

A total of 9 cases of GBM with calcification were included in this study, comprising 4 cases diagnosed in our institution and 5 cases reported in the literature, with an incidence of 0.95%. The median age at diagnosis was 47 years, with a male-to-female ratio of 4:5. Imaging findings were mainly supratentorial lesions, predominantly showing long T1 and long T2 signals, with calcifications appearing as punctate or linear patterns accompanied by significant peritumoral edema, necrosis, and cystic changes. Histologically, all cases demonstrated typical GBM features, with calcification observed in some specimens. Immunophenotypically, Ki-67, Olig-2, and P53 showed high positivity rates.

**Interventions:**

Patients who underwent gross total resection had longer survival times than those who underwent partial resection.

**Outcomes:**

In terms of prognosis, the median postoperative overall survival time of GBM patients with calcification was 91.32 days, significantly shorter than that of general GBM patients in the same period (570 days). These findings suggest that calcification may be a potential indicator of poor prognosis in GBM.

**Lessons:**

The clinical manifestations of GBM patients with calcification have certain characteristics, and the impact of surgery on these patients is different from that on non-calcified GBM. The timing of surgery needs to be carefully selected.

## 1. Introduction

Glioblastoma (GBM) represents the most prevalent, primary, and malignant tumor in the brain.^[1]^ The tendency to calcification is known to be more common in benign tumors and is more frequent in oligodendrogliomas, ventricular meningiomas, and benign astrocytomas.^[2]^ GBM combined with calcification is a clinically rare type that has been less frequently reported in the literature. Clinical treatment and prognosis are unclear, and a retrospective study of its clinical features and treatment strategies is necessary. We followed up patients with pathologically confirmed GBM combined with calcification in our hospital in recent years and analyzed their clinical features and molecular pathology and their prognosis in the light of literature reports. To the best of our knowledge, detailed information on only 5 patients with GBM combined with calcification is available in the English literature, and we summarized the cases in the relevant literature to improve the understanding of this type of glioma and to explore rational treatment strategies.

## 2. Materials and methods

### 2.1. Patient inclusion

The review of patients in the database was approved by the Hospital Review Board. A retrospective evaluation of patients with GBM who underwent surgery-based treatment was performed. Inclusion criteria: patients with a complete medical history; cases showing calcification on imaging. Exclusion criteria: patients whose calcification appeared after treatment; no clear treatment information was available. In the Pubmed website, the terms “glioblastoma[title]” and “calcification” were used as the search term to search literature, and a total of 26 relevant papers were retrieved, and 5 patients (patient number 5–10) were included.^[3-7]^

### 2.2. Clinical study indicators and follow-up

The clinical research parameters included patient sex, age, imaging findings, extent of surgical resection, pathological features, and postoperative treatment. Follow-up was conducted for all enrolled patients (patient number 1–4) through outpatient visits or telephone interviews, with follow-up ending on May 1, 2025.

### 2.3. Tumor histological characteristics and molecular pathological analysis

Immunohistochemistry markers tested included: *ATRX* (X-linked alpha thalassemia mental retardation syndrome), *Ki-67*, *CD34*, *P53*, epithelial membrane antigen (*EMA*), *Olig-2*, *IDH1* (isocitrate dehydrogenase 1), *D2-40* (podoplanin), and Calretinin (CR). Molecular pathology analysis comprised *IDH1/2* mutation testing and *MGMT* (O6-methylguanine-DNA methyltransferase) promoter methylation status.

### 2.4. Statistical methods

R version 4.3.2 was used for data entry and processing. Overall survival was defined as the time from the first symptom or imaging finding to death, and censored data were recorded for patients who were alive at the final follow-up. Statistics were analyzed using descriptive statistics.

## 3. Case information

**Figure 1.**
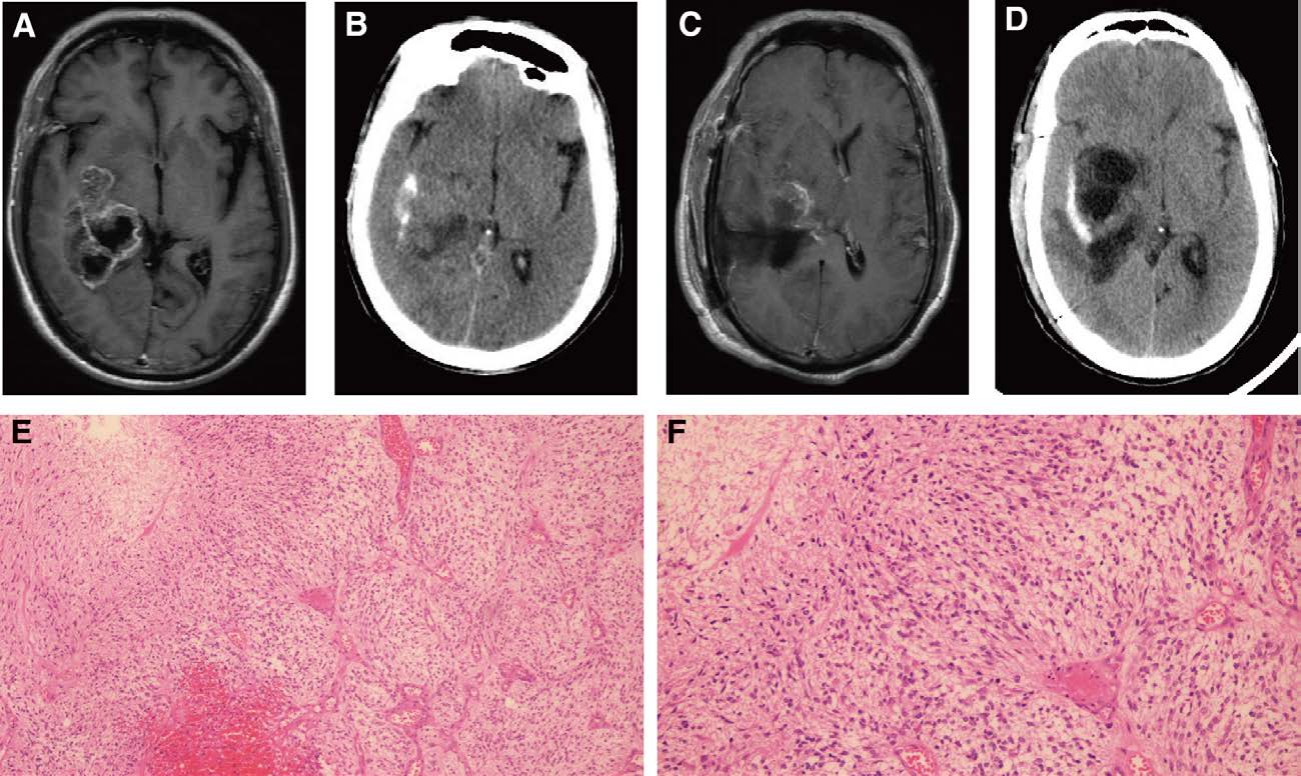
Clinical data of patient number 1. (A) MRI shows patchy hypodense lesions in the right temporal insular lobe, basal ganglia, and corpus callosum, with ring enhancement. (B) CT shows cord-like calcification in the right temporal lobe. (C) Postoperative MRI indicates residual enhancing lesions in the basal ganglia region, with midline structures shifted to the left. (D) Postoperative CT shows residual cord-like calcification in the right temporal lobe. (E, F) Pathology shows spindle-shaped/oval-nuclei tumor cells, with high cellularity and moderate atypia. Pseudopalisading necrosis and glomeruloid microvascular proliferation are observed (left, ×100). Tumor cells exhibit moderate atypia and visible mitotic figures, along with pseudopalisading necrosis and glomeruloid microvascular proliferation (right, ×200). CT = computerized tomography, MRI = magnetic resonance imaging.

Patient number 1, male, 58 years old, presented with headache for 1 week. Cranial MRI indicated a space-occupying lesion involving the right temporal insular lobe, basal ganglia, and corpus callosum (Fig. 1A). CT revealed cord-like calcification (Fig. 1B). The patient underwent gross total resection of the tumor via craniotomy. Postoperative MRI and CT are shown in Figure 1C, D, respectively. Pathological diagnosis confirmed GBM, WHO grade 4 (Fig. 1E, F). Immunohistochemical results were *ATRX* (+), *Ki-67* (+), *CD34* (+), *P53* (−), *EMA* (+), *Olig-2* (+), *D2-40* (+), and *IDH* (−). The patient died on postoperative day 21.

**Figure 2.**
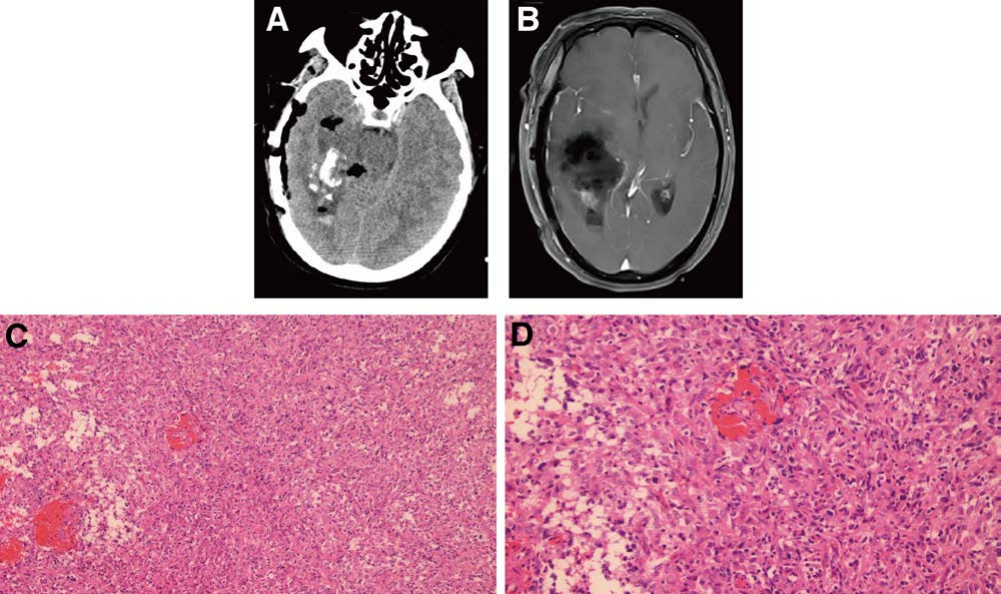
Clinical data of patient number 2. (A) Postoperative operative CT shows residual punctate and patchy calcification foci. (B) Postoperative MRI shows residual patchy enhancing lesions. (C, D) Pathology shows: tumor composed of gemistocytic astrocytes, with increased cellularity and marked atypia; microvascular proliferation is observed, with no definitive tumor necrosis (left, ×100). Gemistocytic astrocytes exhibit significant atypia, with visible mitotic figures and microvascular proliferation; no definitive tumor necrosis is observed (right, ×200). CT = computerized tomography, MRI = magnetic resonance imaging.

Patient number 2, female, 62 years old, presented with intermittent headache and dizziness for 1 year. Cranial MRI indicated an occupying lesion in the right ventricular region, suggesting a possible glioma with localized hemorrhage (preoperative imaging unavailable). Postoperative CT revealed a residual calcified focus (Fig. 2A), and postoperative MRI showed the condition after lesion resection as illustrated in Figure 2B. The patient underwent gross total resection of the tumor via craniotomy. Postoperative pathology indicated a high-grade glioma of the right frontal lobe, and based on tissue morphology and immunohistochemistry, it was consistent with GBM, WHO grade 4 (Fig. 2C, D). Immunohistochemical results were *Ki-67* (+), *CD34* (+), *P53* (+), *Olig-2* (+), and *IDH* (+). The patient received postoperative radiotherapy combined with temozolomide chemotherapy and died 38 months after surgery.

**Figure 3.**
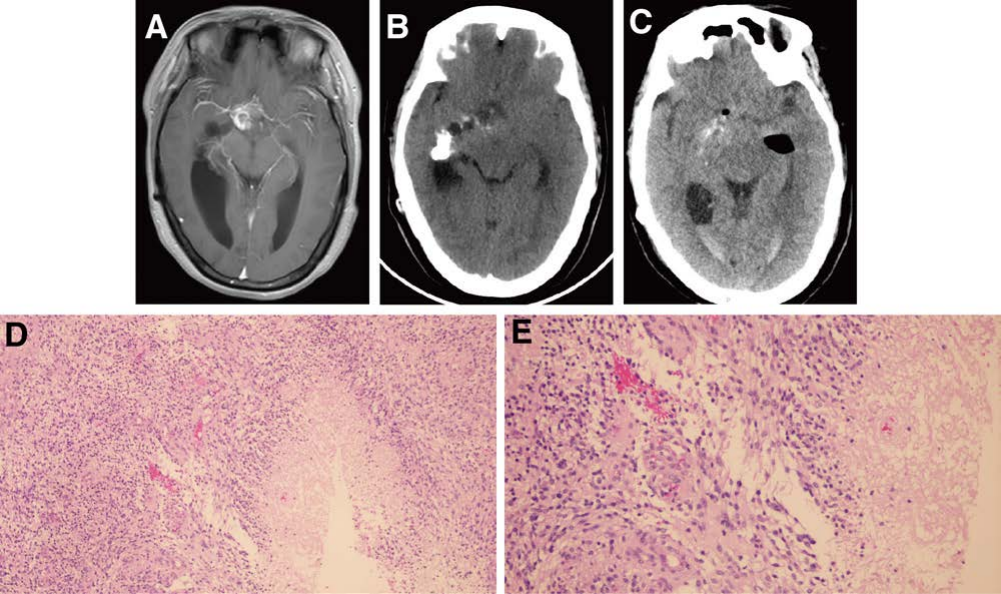
Clinical data of patient number 3. (A) an irregular, patchy isointense T1 and slightly hyperintense T2 signal was observed in the right lateral ventricle and septum pellucidum-foramen of Monro region. The signal was heterogeneous; FLAIR showed uneven hyperintensity, DWI showed high signal intensity, and ADC showed low signal intensity. Contrast-enhanced imaging demonstrated markedly heterogeneous enhancement of the lesion. (B) Preoperative CT revealed irregular, mass-like mixed hyperdense and hypodense lesions in the midline region between bilateral lateral ventricles, within the right lateral ventricle, basal ganglia, and right temporal insular lobe. The lesion margins were ill-defined, with intralesional clump-like calcification and cystic changes. (C) Postoperative CT showed irregular, mass-like mixed hyperdense and hypodense lesions in the midline region between bilateral lateral ventricles, within the right lateral ventricle, basal ganglia, and right temporal insular lobe. The lesion margins remained ill-defined, with clump-like calcification and cystic changes. (D, E) Tumor cells with oval nuclei and high cellularity showed moderate atypia. Pseudopalisading necrosis and microvascular proliferation were observed (left, ×100). Tumor cells demonstrated moderate atypia with visible mitotic figures, along with pseudopalisading necrosis and glomeruloid microvascular proliferation (right, ×200). ADC = apparent diffusion coefficient, CT = computerized tomography, DWI = diffusion weighted imaging, FLAIR = fluid attenuated inversion recovery.

Patient number 3, female, 32 years old, was found to have an intracranial space-occupying lesion 15 years ago and had intermittent headaches for the past year. Cranial MRI indicated a frontal lobe mass with ill-defined borders, obvious calcification within the tumor, and heterogeneous enhancement on contrast scan (Fig. 3A). Preoperative and postoperative CT are shown in Figure 3B, C. The patient underwent partial resection of the tumor via craniotomy. Pathology revealed a diffuse high-grade glioma with pseudopalisading necrosis and glomeruloid microvascular proliferation. Based on histological features, immunohistochemistry, and molecular pathology, the diagnosis was *IDH*-wildtype GBM, CNS-WHO grade 4 (Fig. 3D, E). Immunohistochemical results were *ATRX* (+), *Ki-67* (+), *CD34* (−), *P53* (+), *EMA* (−), *Olig-2* (+), *CR* (−), *D2-40* (−), *MGMT* (−), and *IDH* (−). The patient died from brain herniation on postoperative day 24.

**Figure 4.**
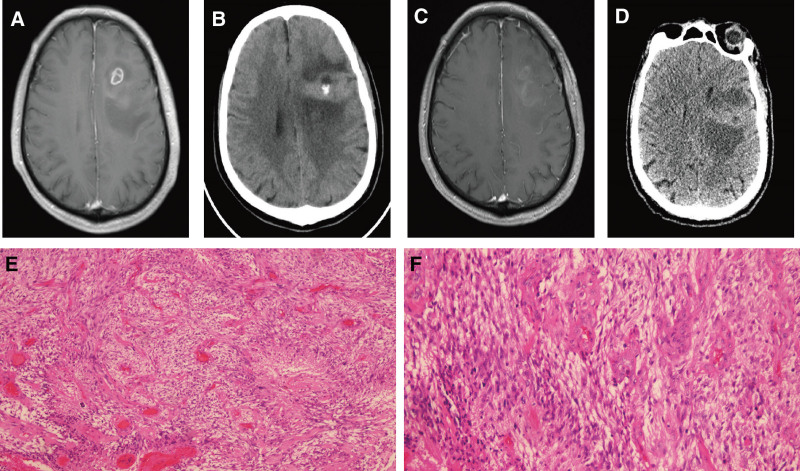
Clinical data of patient number 4. (A) preoperative MRI shows a cystic solid enhancing lesion in the frontal lobe. (B) CT shows a nodular high density lesion in the left frontal lobe. (C, D) Postoperative images show that the majority of the tumor was resected. (E, F) Diffuse glioma characterized by abundant tumor cells, marked cellular atypia, visible mitotic figures, palisading necrosis, and glomeruloid microvascular proliferation. Tumor cells exhibited diffuse growth with high cellular density (left, ×100). Focal areas showed marked cellular atypia, accompanied by necrosis and vascular proliferation (right, ×200). CT = computerized tomography, MRI = magnetic resonance imaging.

Patient number 4, male, 67 years old, presented with a 1-month history of memory decline and slowed responses. Cranial MRI indicated a frontal lobe mass with ill-defined margins and obvious calcification within the tumor. Contrast-enhanced imaging showed heterogeneous enhancement (Fig. 1A). Preoperative and postoperative CT and MRI are shown in Figure 1B–D. The patient underwent partial resection of the tumor via craniotomy. Pathology indicated a high-grade glioma in the left frontal lobe with necrosis and vascular endothelial proliferation. Based on immunohistochemistry and histomorphology, the diagnosis was consistent with *IDH*-wildtype GBM, CNS-WHO grade 4 (Fig. 4E, F). The tumor cells showed diffuse growth with high cellular density, and focal regions exhibited marked cellular atypia, necrosis, and vascular proliferation. Immunohistochemical results were *ATRX* (+), *Ki-67* (+), *CD34* (+), *P53* (+), *EMA* (−), *Olig-2* (+), *CR* (−), *D2-40* (+), *MGMT* (−), and *IDH* (−). The patient received postoperative radiotherapy and temozolomide chemotherapy. Disease progression was noted on postoperative day 85, and the patient died on day 261 due to disease deterioration.

## 4. Results

### 4.1. Clinical features

Among 421 GBM patients reviewed during the same period, 4 cases met the criteria for GBM with calcification, with an incidence of 0.95%. Additionally, 5 cases were included from the literature review, resulting in a total of 9 cases (patient numbers 1–9). The median age at diagnosis of GBM was 47 years, with a male-to-female ratio of 4:5. All 9 patients underwent successful tumor resection.

Among the 4 pathologically confirmed GBM patients included in our study, the average age at presentation was 54.75 years, with a male-to-female ratio of 1:1. Two patients underwent gross total tumor resection, while the other 2 underwent partial resection. Postoperatively, 2 patients received concurrent radiotherapy and chemotherapy.

A total of 5 previously reported cases of GBM were included from the literature, with an average age at presentation of 42 years and a male-to-female ratio of 2:3. Among them, 3 patients underwent partial resection and 2 underwent gross total resection. Postoperatively, 2 patients received radiotherapy only (Table 1).

**Table 1:** Clinical features of 9 cases of GBM with calcification.

Patient number	Author/year	Age at diagnosis (yr)	Sex	Medical history/main symptoms	Tumor location	Extent of resection	Radiotherapy	Chemotherapy	Disease course	Postoperative survival	Cause of death	Tumor recurrence/progression
1	Present/2024	58	Male	Right frontal pain	Right temporo-occipital lobe	Total resection	No	No	7 d	21 d	Unknown	No
2	Present/2024	62	Female	Headache, dizziness	Right temporal lobe	Total resection	Yes	Yes	1 mo	38 mo	Unknown	No
3	Present/2024	32	Female	Intracranial space-occupying lesion for 15 yr, intermittent headache	Right lateral ventricle	Partial resection	No	No	180 mo	24 d	Disease deterioration	No
4	Present/2024	67	Male	Memory impairment, slow response	Left frontal lobe	Partial resection	Yes	Yes	1 mo	261 d	Disease deterioration	Yes
5	Kubota, T/1986^[3]^	27	Female	Numbness in right fingertips	C-3 level intramedullary	Partial resection	Yes	Unknown	1 yr	4 mo	Disease deterioration	No
6	Miyahara, Makiko/2016^[4]^	42	Male	Headache and aphasia	Left parietal lobe	Total resection	No	No	1.5 mo	3 mo	Metastasis	Yes
7	Nitta, Naoki/2018^[5]^	47	Female	Headache, nausea	Right ventricle	Partial resection	No	No	3 yr	33 d	Brain death	No
8	Mallya, V/2015^[6]^	42	Male	Seizures, headache, vomiting	Left frontoparietal region	Total resection	No	No	3 mo	17 mo	Unknown	Unknown
9	Victor, S/1993^[8]^	52	Female	Seizures	Right parietal lobe	Partial resection	Yes	Unknown	10 mo	1 mo	Acute gastrointestinal bleeding	Unknown

GBM = glioblastoma.

### 4.2. Image characteristics

**Figure 5.**
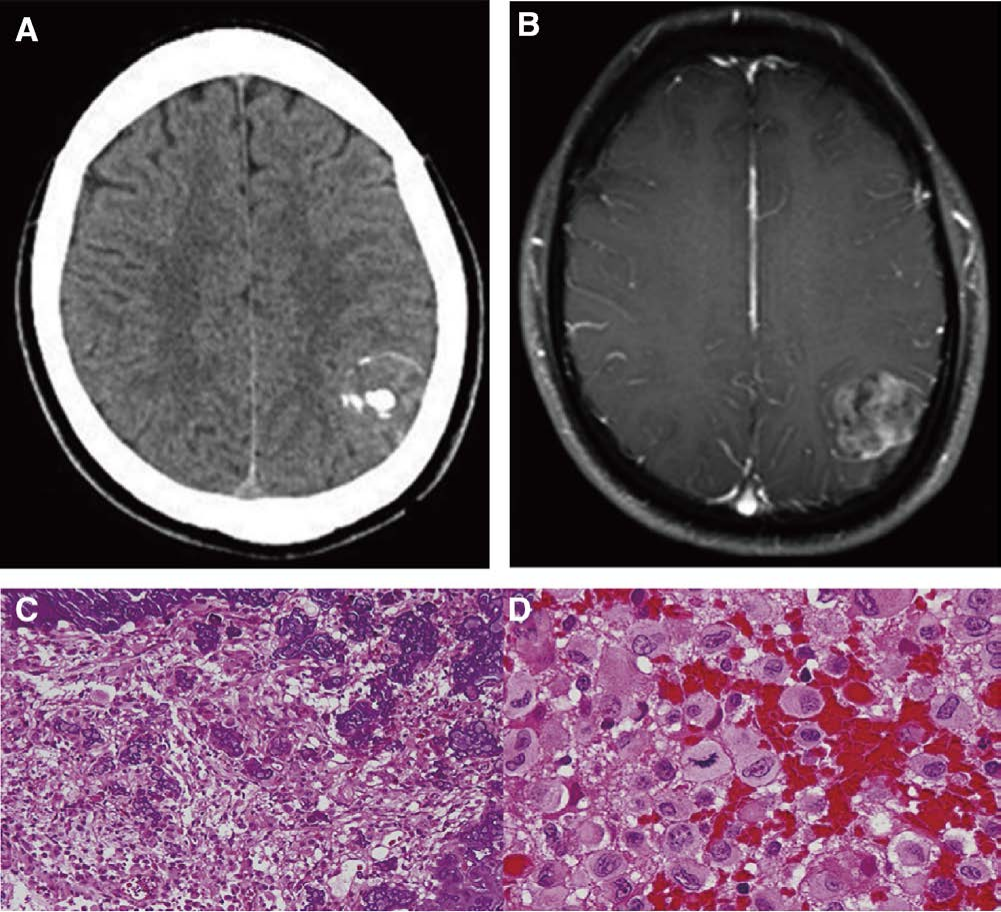
Clinical data of patient number 6. (A) Computed tomography on admission showing a calcific lesion (2.2 cm) in the left parietal lobe. (B) T1-weighted magnetic resonance image after administration of gadolinium-diethylenetriamine pentaacetic acid showing a slight hemorrhage in the tumor and strong enhancement of the tumor. (C, D) Micrograph of a tissue section from the surgical specimen. The large round cell is a rhabdoid cell that has an eosinophilic inclusion in the cytoplasm. Some components resembling low-grade glioma with calcification are present (Source: Ref.^[4]^).

CT and MRI images from different time points for the 4 patients showed that most lesions were located supratentorially, such as in the frontal, temporal, and occipital lobes. The lesions were primarily characterized by long T1 and long T2 signals, with scattered areas of long T1 and short T2 signals. Calcified regions were found within or around the tumors. On CT, the calcifications appeared as multiple punctate or linear high-density areas. Perilesional edema was evident, and on MRI, these regions exhibited long or slightly long T1 and short T2 signals. Imaging features included necrosis, cystic degeneration, edema, poorly defined margins, and abnormal vasculature, as shown in patient number 4 (Fig. 4A–D). The lesion locations in this group of patients were also consistent with those reported in the literature, most commonly involving the frontal and temporal lobes. Imaging signals also showed a high degree of consistency, as exemplified by patient number 6 (Fig. 5A, B).

### 4.3. Pathological characteristics

The histological specimens possessed by the 10 patients were mostly gray, gray-yellow or gray-red, with soft texture, and hemorrhage, necrosis and calcification were seen in some specimens. Microscopically, they had features of typical necrosis and heterogeneous cells in the GBM lesion area, irregular cell morphology, visible microvascular proliferation, and some of them clearly suggested calcification in the tumor area (Fig. 2C). Patient numbers 1 to 4 immunophenotypes were accompanied by different molecular features, such as *IDH* wild type or mutant, *Ki-67* expression levels, and other molecular markers. The immunomarkers in descending order of the incidence of positivity were *Ki-67* (4/4), *CD34* (3/4), *Olig-2* (4/4), *D2-40* (2/3), *P53* (3/4), *IDH* (1/4) *MGMT* (0/2; Table 2). Among the typical cases were patient number 4 (Fig. 1E) and patient number 6 (Fig. 2C). The histological morphology of the tumors showed less pleomorphism and more orderly cellular arrangement compared to conventional GBMs.

**Table 2:** Molecular pathological characteristics of the 4 GBM patients with calcification.

Patient number	ATRX	Ki-67	CD34	P53	EMA	Olig-2	CR	D2-40	MGMT	IDH
1	+	+	+	−	+	+	None	+	None	−
2	None*	+	+	+	None	+	None	None	None	+
3	+	+	−	+	−	+	−	−	−	−
4	+	+	+	+	−	+	−	+	−	−

ARTX = X-linked alpha thalassemia mental retardation syndrome, CR = calretinin, EMA = epithelial membrane antigen, GBM = glioblastoma, IDH = isocitrate dehydrogenase, MGMT = O6-methyguanine-DNA methytransferase.

* “None” indicates that the relevant pathology has not been reported.

In the pathological descriptions from the literature, no significant differences in histological morphology of the tumors were observed. Among them, calcification within the tumor area was explicitly mentioned in the pathological report of patient number 6 (Fig. 5C, D). It is considered that due to limited awareness of calcification in GBM, specimen collection may have focused only on the softer tumor tissue. Unfortunately, the reported cases in the literature lacked descriptions of molecular features and immunohistochemical markers in the pathology.

### 4.4. Prognosis

Follow-up data showed that for patient numbers 1 to 4, the average postoperative survival time was 588.86 days for patients who underwent gross total resection, and 142.5 days for those who underwent partial resection. The overall average postoperative survival time was 365.68 days.

For the 5 patients included from the literature, the average postoperative survival time after gross total resection was 304.4 days, and 61.73 days after partial resection. The overall average postoperative survival time was 158.8 days.

From the follow-up data, gross total resection is associated with better prognosis, but overall postoperative survival remains poor.

Overall survival was defined as the time from the first onset of symptoms or detection by imaging to death, with patients who were still alive at the last follow-up recorded as censored data. A total of 109 conventional GBM patients treated at our institution during the same period were followed, among whom data were missing for 53 cases. For the remaining 56 patients, overall survival ranged from 47 days to 147 months, and postoperative survival ranged from 28 days to 87 months. The median postoperative survival was 570 days, which was significantly longer than the median survival of 91.32 days observed in the 9 GBM patients with calcification.

## 5. Discussion

GBM is a common primary malignant tumor of the brain, whereas GBM with calcification is rare. As the largest study to date covering the number of cases with a detailed course in patients with GBM combined with calcification, we found that the incidence of patients with GBM combined with calcification was 0.95%. This is lower than the 7% reported in 1977,^[2]^ We considered that it might be limited by the classification of gliomas at that time and the lack of knowledge about GBM. GBM is a disease with a higher incidence in females. In both the cases reported in the literature and those collected in our study, the ratio of males to females with calcification is approximately 1:1. Moreover, our findings suggest that calcification may not be associated with gender or geographical factors. Moreover, the lower median age at diagnosis observed in calcified GBM patients compared to the median diagnosis age of 65 years for conventional GBM suggests that tumor calcification may be associated with a distinct clinicopathological subtype.^[8]^ Patients such as case numbers 3, 5, 7, and 9 were relatively young and exhibited a prolonged preoperative disease course. This particular subtype is characterized by younger onset, a less aggressive clinical course, and slower tumor progression – biological features that may collectively create a microenvironment conducive to calcification development.

The standard treatment for GBM includes surgery and postoperative simultaneous radiotherapy.^[9]^ However, the low rate of radiotherapy in our group is, we believe, related to the poor postoperative prognosis of the patients, for example, patient number 3 and number 7, who died within a very short period of time after surgery.

In imaging findings, although calcification can indicate tumor location, the precise position, morphology, and extent of calcium deposition provide little definitive diagnostic evidence and show no correlation with tumor size.^[2]^ However, for calcifications due to other factors, certain characteristics remain, such as Teo, JG et al who found a vascular distribution of calcified foci in patients with a history of lead poisoning.^[10]^ We were unable to draw meaningful conclusions about the location of calcifications due to limitations in the number of cases. However, we should still be vigilant that intracranial occupations presenting with calcifications should not exclude the diagnosis of GBM to avoid delays in treatment.

Regarding the pathologic features of GBM, all of our cases *IDH* tend to be wild type, which is an important change in the 2021 GBM classification.^[11]^ Since the latest classification of gliomas removes the classification of secondary GBM, this is one of the reasons for the low rate of GBM combined with calcification that we reported. In addition, our pathological findings showed that calcification was not mentioned in the pathology reports of all 4 patients, which may be related to the fact that the calcified lesions were not resected. In their study, Kalan, C et al found that in some patients, although the amount of calcium deposition was insufficient to form visible x-ray imaging, calcification was observed under microscopic examination,^[12]^ suggesting that the actual proportion of calcification may be higher. The presence of calcifications has been shown to be associated with a better prognosis in patients with GBM, as observed in patient number 2. However, the relationship between the extent of calcification and survival requires further investigation.^[2]^

Among the 9 patients who underwent surgery, the median duration of disease before surgery was 3 months, which is longer than the median preoperative course of 40 days for typical GBM,^[13]^ suggesting a potentially better prognosis. However, the median postoperative survival was 91.32 days, with 5 patients surviving for only a very short period after surgery. This is significantly lower than the median postoperative survival of 570 days observed in the 109 patients we followed. Excluding surgeon-related factors, we believe that for GBM with calcification, the decision to proceed directly with surgical resection should be made cautiously. Stereotactic biopsy may be considered for diagnosis, followed by preoperative radiotherapy to improve prognosis.^[14]^

Relevant literature suggests that calcification may result from lead poisoning. When considered as a potential etiological factor in GBM formation, lead poisoning may cause primary vascular injury followed by secondary dystrophic calcification. This type of calcification presents a unique pattern in which only the vasculature is involved, without calcification of the brain parenchyma. On CT imaging, curvilinear and punctate calcification patterns can be observed, which are associated with characteristic histological features.^[10]^ Kubota et al propose that calcified areas exist in gliomas with histologically benign features and hypothesize that these calcified regions within originally benign astrocytomas may undergo either gradual progression or abrupt malignant transformation, ultimately evolving into GBM.^[3]^ In the study by Teo, JG et al due to the patient’s asymptomatic period of up to 8 months and the long term stability of the CT calcified foci, it was likewise hypothesized that the tumor was a secondary GBM, which did not originate from primitive undifferentiated cells, but rather evolved from a primary astrocyte component or an oligodendrocyte component that underwent progressive secondary dedifferentiation, a hypothesis which was supported by histological examination: a significant nuclear pleomorphism was seen in the pathological description of astrocytic proliferation.^[7]^ However, in a study by Kalan, C et al, the morphology of the calcium deposits and the extent of the deposits did not seem to be related to the degree of malignancy of the tumor, and it was almost impossible to differentiate between calcifications in gliomas and other brain tumors (e.g., meningiomas) based on the morphology alone or to distinguish intracranial calcified foci from neoplastic or nonneoplastic ones.^[12]^ Takeuchi, K et al also suggested that even though calcification takes longer to form, there is no contradiction between the presence or absence of calcification and survival in malignant tumors with a very short prognosis, such as GBM.^[2]^ Meanwhile, according to the embryogenesis theory (which advocates that tumors originate from abnormal differentiation of immature cells, and that different brain tumor types correspond to neuroepithelial stem cells with different degrees of differentiation), it is suggested that GBM may have originated from undifferentiated glial cells (i.e., the tumor cells do not have clear features of astrocyte or oligodendrocyte differentiation).^[7]^ Therefore, based on the patient’s pathology report, we believe that the calcifications could have originated directly from the *IDH* wild type GBM rather than evolving from one of the lower grade tumors. This is also supported by the 2021 glioma classification, which designates GBM specifically as *IDH*-wildtype glioma, with what was previously considered secondary GBM now classified as *IDH*-mutant astrocytoma.^[11]^

Current reports on the formation of calcification in GBM may be related to lead poisoning, neurofibromatosis type 1 (NF 1), fragile X chromosome syndrome, and *FGFR3::TACC3* fusion, etc. Takeuchi, K et al suggested that if a way to accelerate calcium deposition in tumors can be found, it may provide a direct or indirect therapeutic approach to extend the survival of GBM patients.^[2,10,15-17]^

Among the 9 patients who underwent surgery, the median survival time postoperatively was 90 days, with 5 patients surviving for a very short period after surgery. This is significantly lower than the median postoperative survival time of 570 days in the 109 patients we followed during the same period. Given that patients with GBM and calcification are generally expected to have a relatively favorable prognosis, we suggest that the surgical approach for GBM with calcification should be considered carefully. The goal should be to achieve a clear diagnosis and reduce tumor burden, avoiding aggressive resection when possible. For some patients, stereotactic biopsy followed by radiotherapy and chemotherapy may be considered after a definitive diagnosis.

In summary, calcification is often considered a potential favorable prognostic factor in GBM patients. However, in the cases included in this study, the postoperative outcomes of GBM patients with calcification were not superior to those of common GBM and instead showed a certain adverse trend. We speculate that possible reasons include: in some cases, the imaging appearance led to an initial misdiagnosis as a benign lesion, resulting in delayed treatment; in some cases, an overly aggressive surgical timing adversely affected the prognosis. Therefore, we believe that the surgical benefits for such patients are different from those with non-calcified GBM, and it may be a more appropriate treatment strategy to administer radiotherapy and chemotherapy first after a confirmed diagnosis by biopsy. It should be emphasized that this study is a small-sample case series, and the mechanisms by which surgery affects survival in these patients remain unclear, so the conclusions are still limited. Thus, further case accumulation and in-depth multi-center studies are needed to clarify the role of surgery in GBM patients with calcification and to explore more rational diagnostic and therapeutic strategies to improve patient outcomes.

## Author contributions

**Conceptualization:** Chunxiao Ma.

**Data curation:** Mengda Li, Yongji Guo.

**Investigation:** Guanzheng Liu, Xingyao Bu.

**Methodology:** Juntao Li, Zhixiao Li.

**Software:** Juntao Li, Zhixiao Li.

**Validation:** Juntao Li, Zhixiao Li.

**Visualization:** Guanzheng Liu, Xingyao Bu.

**Writing – original draft:** Mengda Li, Yongji Guo.

**Writing – review & editing:** Chunxiao Ma.
